# Allies or Enemies—The Multifaceted Role of Myeloid Cells in the Tumor Microenvironment

**DOI:** 10.3389/fimmu.2019.02746

**Published:** 2019-11-28

**Authors:** Lisa Haas, Anna C. Obenauf

**Affiliations:** Research Institute of Molecular Pathology (IMP), Vienna Biocenter (VBC), Vienna, Austria

**Keywords:** immunotherapy, cancer, myeloid cells, dendritic cells, macrophages, myeloid-derived suppressor cells, immune suppression, tumor microenvironment

## Abstract

For decades, cancer was considered a disease driven by genetic mutations in tumor cells, therefore afflicting a single cell type. This simplified view was slowly replaced by the understanding that interactions between malignant cells and neighboring stromal and immune cells—the tumor microenvironment (TME)—profoundly shape cancer progression. This understanding paved the way for an entirely new form of therapy that targets the immune cell compartment, which has revolutionized the treatment of cancer. In particular, agents activating T lymphocytes have become a key focus of these therapies, as they can induce durable responses in several cancer types. However, T cell targeting agents only benefit a fraction of patients. Thus, it is crucial to identify the roles of other immune cell types in the TME and understand how they influence T cell function and/or whether they present valuable therapeutic targets themselves. In this review, we focus on the myeloid compartment of the TME, a heterogeneous mix of cell types with diverse effector functions. We describe how distinct myeloid cell types can act as enemies of cancer cells by inducing or enhancing an existing immune response, while others act as strong allies, supporting tumor cells in their malignant growth and establishing an immune evasive TME. Specifically, we focus on the role of myeloid cells in the response and resistance to immunotherapy, and how modulating their numbers and/or state could provide alternative therapeutic entry-points.

## Introduction

**Figure 1:**
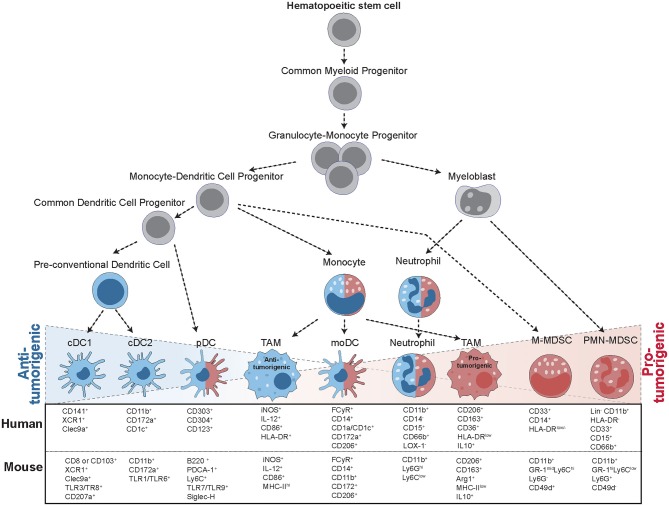
Progression from hematopoietic stem cells (HSC) to tumor-infiltrating myeloid cells. The formation of tumor-infiltrating myeloid cells occurs in a step-wise process: In the bone marrow, HSCs give rise to common myeloid progenitors (CMP), which give rise to granulocyte-monocyte progenitors (GMP). GMPs then further specify into myeloblasts (MB) and monocyte-dendritic cell progenitors (MDP). These precursors then differentiate into a range of different cell types with anti-tumorigenic (blue) and pro-tumorigenic (red) capacities. MDPs can give rise to a common dendritic cell progenitor (CDP), further leading to the formation of conventional DCs (cDCs) or plasmacytoid (pDCs). MDPs also form monocytes, giving rise to monocytic DCs (moDCs) or differentiating into tumor-associated macrophages (TAMS) upon instruction by the tumor. Macrophages can display multiple different activation states, ranging from anti-tumorigenic to pro-tumorigenic subsets. MBs give rise to mature neutrophils or polymorphonuclear myeloid-derived suppressor cells (PMN-MDSCs), while monocytic MDSCs (M-MDSCs) arise from MDPs upon instruction by inflammatory signals. All of these cell types are characterized by specific surface marker expression, indicated for both human and mouse cells.

**Figure 2:**
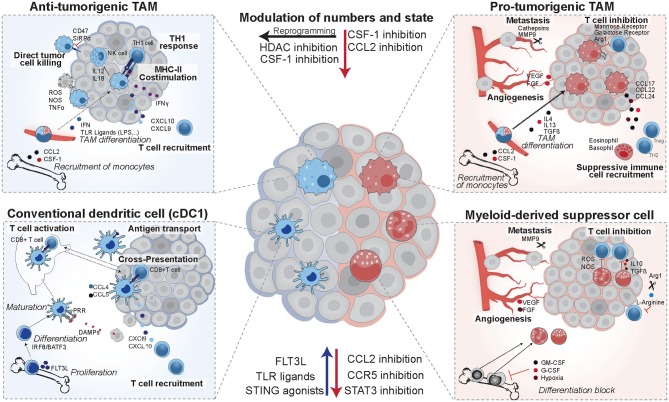
Opposing functions of tumor-infiltrating myeloid cells. *Anti-tumorigenic TAMs* arise from circulating monocytes in response to TLR ligands and interferon. They are characterized by high expression of costimulatory molecules and MHCII. In mouse models they were shown to induce potent TH1 responses and augment NK cells responses. *cDC1* dendritic cells differentiate in response to FLT3L, mature upon recognition of danger associated molecular patterns (DAMPs), and then induce T cell activation via antigen presentation on MHCI. They establish a favorable cytokine environment in the tumor (CXCL9, CXCL10) and murine studies revealed that they are recruited in response to CCL4 and CCL5. In patients, they have positive prognostic value, correlate with T cell infiltration and are enriched in immunotherapy responders. Their numbers and maturation state can be enhanced by FLT3L, TLR ligands, or STING agonists. *Pro-tumorigenic TAMs* arise from circulating monocytes in response to IL4, IL13, and TGFβ, and establish an immune suppressive environment via recruitment of eosinophils, basophils, Tregs, and TH2 cells. They are pro-metastatic and induce angiogenesis, and their recruitment can be reduced by CSF-1 and CCL2 inhibitors in pre-clinical models. In addition, mouse models identified that they can be re-educated to an anti-tumorigenic state using HDAC inhibitors. *MDSCs* form from immature myeloid progenitors upon stimulation by the tumor and suppress T cell activity via IL10, TGFβ, and production of reactive oxygen and nitrogen species (ROS and NOS). They deplete intracellular L-arginine pools and hamper T cell proliferation in murine models and in patients their presence is a negative prognostic factor.

Myeloid cells are a diverse group of cells belonging to the innate immune system that are prone to adapt their phenotype to their tissue of residence (1). Thus, in cancer, they exist in a vast amount of different states and exert a range of distinct functions (Figure 1). Among those myeloid cells, macrophages, dendritic cells (DCs), and myeloid-derived suppressor cells (MDSCs) have received much attention in the last decades, due to their ability to both initiate or suppress an anti-tumor immune response (Figure 2) (2). In the following, we will specifically focus on those three groups of myeloid cells and provide an overview of their roles as cancer cell allies or enemies.

## Dendritic Cells

Since their identification in mice in 1973 by Steinman and Cohn, DCs have become widely accepted as important players in the network of phagocytizing and antigen presenting cells (APCs) that sculpt immune outcomes (3). In tumor immunity, DCs have predominantly an anti-tumorigenic role. DCs arise from a common bone marrow (BM) progenitor—the common dendritic cell progenitor (CDP)—and then differentiate into plasmacytoid (pDCs) and precursors for conventional dendritic cells (cDCs) (Figure 1). These immature DCs subsequently migrate out of the bone marrow and colonize peripheral tissues, where they encounter antigens (4–8). The maturation of DCs represents a critical step in their life-cycle, allowing them to gain full APC capacities. Maturation is initiated upon recognition of danger-associated molecular patterns (DAMPs) via pattern recognition receptors (PRRs), where different DC subsets express different PRRs, further contributing to their functional specification. Upon maturation, DCs upregulate their antigen presentation machinery and costimulatory molecules, transforming themselves into potent T cell activators and thus bridging innate and adaptive immunity (9, 10). DCs can license anti-tumor immune responses by processing and cross-presenting exogenous antigens via MHC class I molecules to CD8 T cells, presenting antigens via MHC class II molecules to CD4 T cells, and secreting immune-stimulatory cytokines. In this capacity, they have become an integral part of the cancer immunity cycle and are attractive targets for immunotherapy (11, 12).

### cDCs Are Potent Activators of Anti-tumor Immunity

cDCs differentiate into two subsets—cDC1 and cDC2—which are distinguished by their differential marker expression (Figure 1), transcription factor (TF) dependency, and functions. The differentiation into cDC1s or cDC2s is instructed by different chemokines and single cell sequencing studies in mice revealed distinct gene signatures that become evident early after the differentiation from CDPs (Figure 1): cDC1s are instructed by FLT3L and express the TFs IRF8, BATF3, and ID2, cDC2s are instructed by GM-CSF and are dependent on the TF IRF4, Notch2, and RelB (4, 8, 13, 14).

The role of cDC1 cells in anti-tumor immunity is well-established (15, 16). cDC1s are present as both lymph node resident (CD8^+^) and migratory (CD103^+^) populations. Lymph node resident DCs sample antigens in blood and lymph fluid, and migratory cDC1s transport antigens from the peripheral tissue to lymph nodes and spleen. This is indicated by the ability of CD103^+^ cDC1s to transport tumor-derived fluorescent proteins to the lymph node in a CCR7-dependent manner (17, 18). A substantial fraction of intratumoral CD103^+^ cDC1s does not migrate to the lymph node, yet they still play a crucial role in anti-tumor immunity. In mouse models those intratumoral, non-migratory CD103^+^ cDC1s were shown to mediate their effects via direct antigen presentation and establishment of a favorable chemokine environment and were found necessary for tumor control in a lymph node-independent manner (13, 17). They are an important source of CXCL9 and CXCL10 in tumors, which makes them indispensable for the infiltration of both naïve and pre-activated T cells. In patients, the levels of CD103^+^ transcripts correlate with the levels of CXCL9 and CXCL10, and degree of T cell infiltration (19, 20). The crucial role of CD103^+^ cDC1s has been further substantiated using BATF3^−/−^ mice devoid of CD103^+^ cDC1s, which fail to reject immunogenic cancer cell lines and are unresponsive to immune checkpoint inhibition (17, 19, 21, 22).

In contrast to cDC1s, the role of the more heterogeneous population of CD11b^+^ cDC2s in anti-tumor immunity is less well-explored. They are superior to cDC1s in the induction of CD4 T cell responses via antigen presentation on MHCII, and have been shown to activate TH17 cells, a cell type with controversial roles in cancer that produces high levels of pro-inflammatory cytokines (23, 24). Compared to cDC1s, cDC2s fail to deliver antigen to lymph nodes. Because they have lower levels of endocytic receptors, higher levels of lysosomal enzymes and a lower phagosomal pH, it was hypothesized that antigens are directly degraded during migration instead of being further processed and presented on the surface (8, 13, 25). However, reduced antigen presentation of cDC2s may (also) be due to a lack of appropriate stimuli in the tumor and if stimulated, cDC2s may still play an important role in anti-tumor immunity. This is supported by studies showing that immune responses induced by the TLR7 agonist R848, acting on cDC2s, or anthracyclines, also induce protection in BATF3-deficient mice (26, 27).

### pDCs and moDCs Have Antagonistic Roles in Cancer Immunity

DCs display high functional plasticity and despite having largely anti-tumorigenic capacities, they can under certain circumstances for example when present in an immature state, act immune suppressive. This is illustrated by the complex role of pDCs in tumor immunity. pDCs express MHCII, costimulatory molecules, and a narrow set of TLR receptors and have been identified as the main producers of Type I IFN upon activation by DAMPs (1, 28). Despite their capacity to produce Type I IFN, the presence of pDCs is a poor prognostic marker in breast cancer, melanoma, and ovarian cancer in human and animal models (29–32). This could be due to the poor activation of pDCs in the TME and an active instruction of pDCs by the tumor to fulfill immune-suppressive functions, such as production of IDO, IL10, or OX40 expression (33). Monocytic DCs (moDCs or inflammatory DCs) have a different origin and differentiate from Ly6C^high^ monocytes in the context of cancer or inflammation (Figure 1). They are efficient in the uptake and processing of antigens and correlate with CD8^+^ T cell infiltration in several tumor models (34). Yet in direct contrast, they can also display an immune-suppressive phenotype, based on high expression of iNOS, TNF-α, IL-6, IL-10, and their capacity to hamper T cell proliferation *in vitro*, as it was shown using moDCs isolated from murine lung cancer models (23, 34). Thus, further investigation is needed to understand the pro- vs. anti-tumorigenic functions of this complex cell type, the tumor-derived signals that skew them, and particularly how this plays out in patient settings.

### Tumors Inhibit DC Functionality on Multiple Levels

In addition to the diverse effects of DCs on tumor cells, in return, the tumors can interfere with DC functionality, either by affecting their differentiation or by suppressing their activation and maturation at the tumor site. Many tumor-secreted factors affect DC differentiation. For example, IL-6 and CSF-1 promote lineage commitment toward suppressive monocytes (35), and vascular endothelial growth factor (VEGF) inhibits DC maturation by suppressing NFκB signaling in hematopoietic progenitors (36). In addition, secreted factors can also directly inhibit the anti-tumor activity of DCs, such as TGF-β, which can inhibit antigen uptake *in vitro* and it was shown that inhibiting TGF-β signaling synergizes with immunotherapy in pre-clinical mouse models (15, 37, 38). In the local TME, metabolic dysfunction can hamper DC activity. For example, high levels of lactic acids were shown to interfere with DC activation and antigen presentation (39). Studies in mouse models showed that lipid peroxidation byproducts can induce continuous activation of the TF XBP1 in DCs, resulting in abnormal lipid accumulation and DC dysfunctionality (40). Recently, it became clear that the TME is strongly influenced by the oncogenic pathways driving cancer progression, which have a profound impact on the immune cell infiltrate (41, 42). In the context of DCs, upregulated beta-catenin signaling reduces infiltration of cDC1s via reducing the production of CCL4, among other chemokines (43). Elevated COX activity in tumor cells results in production of prostaglandin E2, which reduces NK cell infiltration and thus reduced the cDC1 recruitment factors XCL1 and CCL5 in the tumor microenvironment. Consequently, tumors with high prostaglandin E2 displayed reduced cDC1 levels, contributing to reduced effector T cell infiltration (44).

### DCs Promote Response to Immunotherapy

CD8 T cell priming against tumor-specific antigens requires cross-presentation of the antigen on an MHC I complex by DCs and marks a crucial step for mounting a functional T cell response (45). Indeed, the presence of cDC1s in human tumors correlates with T cell infiltration levels and increased survival in breast, lung, and head and neck cancer patients (13). Moreover, murine studies using cDC1-deficient BATF3^−/−^ mice highlighted their crucial importance for the response to immunotherapy (13, 17, 19, 46). Recently, a systematic comparison of biopsies from patients responding vs. non-responding to immunotherapy identified intratumoral abundance of cDC1s (CD141^+^ in humans) as predictive for immunotherapy success (47). This is in line with a second study that characterized IL-12 producing BATF3^+^ DCs as crucial for immunotherapy success in mice and showed that IL-12 activates lymphocyte effector functions in patients (48).

### Targeting DCs as a Therapeutic Strategy

The central role of DCs in the initiation of immunity and their positive effect on patient survival provide a strong rationale to harness DCs and boost an endogenous anti-tumor immune response. To this end, different approaches are being explored, including: (1) increasing intra-tumoral DC numbers, (2) boosting DC maturation and function, and/or (3) alleviating tumor cell-mediated DC repression (8, 49). Vaccination strategies to increase DC numbers using both non-targeting and targeting vaccines represented a first wave of therapies that was initiated more than two decades ago (50). Non- targeting vaccines composed of peptides together with adjuvant agents showed limited clinical success and were later improved to contain patients' antigenic peptides in combination with the chemokine GM-CSF, resulting in clinical responses (51, 52). In addition, GVAX—a vaccine containing cancer cells overexpressing GM-CSF—was shown to attract and activate DCs in patients, and later to have some clinical activity (53). There remains however a big discrepancy between the capacity of these vaccinations to induce DC activation and their actual clinical efficacy. This could be due to a suppressive TME and exhaustion of T cells, and thus combination therapies may be the key to their success and are being actively explored in pre-clinical and clinical studies (12, 50, 53, 54). In 2012, a combination trial of GVAX and checkpoint inhibition was shown to be clinically safe (55) and more recently in 2016, an overall response rate of 38% was achieved in patients receiving transfer of modified, autologous DCs with checkpoint inhibition (56). Intra-tumoral injection of FLT3L increases numbers of circulating cDC1s, mobilizes DCs to the TME and has been successful in murine studies in combination with Poly I:C induced maturation (17). In patients, injections of FLT3L resulted in an increase of circulating cDCs (57).

An alternative approach is the maturation of DCs, which results in high expression of chemokines, costimulatory molecules and antigen presentation (9). Different maturation cocktails, comprising proinflammatory cytokines or TLR ligands have been evaluated in clinics and were shown to induce robust T cell activation capacities in DCs (58). To reduce side effects a direct intra-tumoral administration of maturation stimuli may be preferred and direct and abscopal effects of the TLR ligands Poly I:C or CpG are being evaluated (59). The STING pathway, sensing cytoplasmic DNA and inducing prominent Type I interferon release from DCs, has been another focus of intense research and modified cyclic dinucleotides, mimicking the endogenous STING ligands, have progressed into clinical trials (60). In addition, in 2018 a small molecule STING agonist has been published to induce potent, long-lasting responses in mice bearing colon cancer (61). While many of these approaches focus on cDC1s, triggering the release of IFN by pDCs could be an alternative entry point, which is under active investigation in checkpoint inhibitor resistant melanoma patients (62, 63).

## Tumor-Associated Macrophages (TAMs)

Macrophages are a heterogeneous population of myeloid cells and are highly abundant in many cancer types. Their heterogeneity is influenced by: (1) their developmental origin, (2) their tissue of residence, and (3) the environmental cues they are exposed to (64). This is reflected by the vast number of different activation states, ranging from anti-tumorigenic to strongly pro-tumorigenic phenotypes.

### TAMs Are a Heterogeneous Population of Myeloid Cells With Different Developmental Origins

In tumors, it was predominantly believed that TAMs arise from circulating monocytes that are recruited from the BM or spleen via cytokines such as CCL2 and CSF-1. However, macrophages can also arise from embryonic precursors and develop into tissue-resident macrophages, such as microglia in the brain, alveolar macrophages in the lung, or Kupffer cells in the liver (65, 66). In recent years, it has become clear that both monocyte-derived and tissue-resident macrophages play a role in tumorigenesis. Lineage tracing studies in mouse brain tumors revealed that both tissue-resident and monocyte-derived macrophages populate brain tumors, and macrophages of dual origin were reported in pancreatic ductal adenocarcinoma (67–70). The identification of a unique marker to characterize these heterogeneous TAM populations has proven difficult. In murine macrophages the glycoprotein CD68 is fairly specific and in combination with F4/80 identifies the majority of TAMs. In humans, CD68 is less specific and also expressed on granulocytes, dendritic cells, endothelial cells, fibroblasts and some lymphoid subsets (71). Due to the lack of a specific marker, the scavenger receptor CD163 (in humans M130) is often used in combination with CD68 to identify TAMs in humans (2). Moreover, CD49D can be used as a discriminatory marker between bone-marrow derived macrophages recruited to the brain and tissue-resident microglia in both mouse and humans and CD45 expression levels allow to distinguish these cell types in murine tumors (67).

Due to their substantial heterogeneity, TAMs need further sub-classification. They are commonly divided into “classically activated” M1 and “alternatively activated” M2 macrophages, with M1 referring to anti-tumorigenic and M2 to pro-tumorigenic macrophages. However, this classification is an oversimplification and the M1/M2 activation states present the extremes of a large spectrum of different functional states with various features (72, 73). Pro- and anti-tumorigenic TAMs are instructed by different sets of stimuli: anti-tumorigenic TAMs arise in response to TLR ligands and IFN, whereas pro-tumorigenic TAMs expand in response to IL4, IL13, TGFβ, and glucocorticoids (73–76). TAMs with anti-tumorigenic potential produce IFNγ, have high levels of MHCII and costimulatory molecules and secrete TH1-recruiting chemokines such as CXCL9 and CXCL10. They are strong promoters of TH1 responses, which results in production of IFNγ and IL12, and induces a positive feedback loop. In addition, anti-tumorigenic macrophages augment NK cell responses by producing IL18 and IL22 (Figure 2) (2, 77–79). In contrast, TAMs acting in a pro-tumorigenic manner are more phagocytic, express higher levels of mannose and galactose receptors, and have a highly active arginase pathway (79). The depletion of arginine pools by Arg1, an enzyme converting L-arginine into L-ornithine, is detrimental to T cells and has been shown to drive their cycle arrest in murine models (80, 81). Additionally, pro-tumorigenic TAMs express a distinct set of chemokines, including CCL17, CCL22, and CCL24. This, in turn, recruits TH2 cells, regulatory T cells, eosinophils and basophils, and induces a more immune suppressive microenvironment (76). Bulk sequencing studies of breast and endometrial cancer patient-derived monocytes and TAMs published earlier this year, provided further insight into human TAMs and identified CCL8 as an additional pro-tumorigenic TAM effector molecule, inducing the expression of an invasive gene expression profile in the cancer cells (82).

Moreover, the spatial distribution of macrophages and the respective environmental conditions in different tumor areas has a profound impact on their function. At the leading edge of tumors, macrophages can drive invasive cellular states through a paracrine signaling loop involving CSF-1 and EGF (83). They act as a major source of matrix metalloproteinases, cathepsins, and serine proteases, which promote degradation of basement membranes and promote invasion and metastases (84–86). In growing tumors TAMs frequently accumulate in regions of hypoxia, where the hypoxic conditions could induce a switch to a pro-angiogenic, invasive phenotype, mediated via diverse range of angiogenic factors, such as TGFβ, VEGF, PDGF, and fibrin (83, 87).

### TAM Activation Influences Patient Prognosis and Response to Immunotherapy

High levels of TAMs are associated with poor prognosis, such as in patients with breast, lung, head and neck cancer, as well as Hodgkin's lymphoma. However, high levels of CD68^+^ cells (consisting largely of TAMs but also granulocytes, dendritic cells and fibroblasts, which also express this marker) are reported to correlate with better prognosis in patients with colon, gastric, and endometrial cancer (2, 71, 88, 89). In consideration of the vast heterogeneity of this cell type, the activation state of TAMs may be a better prognostic marker than cell numbers. Especially the strong immune-suppressive effects of pro-tumorigenic TAMs and their expression of PD-L1, PD-L2, CD80, and CD86, which are ligands for the T cell checkpoints PD-1 and CTLA-4, would suggest TAM infiltration to have a negative effect on immunotherapy. Indeed, in several studies using mouse models, depletion or re-education of TAMs using HDAC inhibitors or blockade of CSF-1 signaling, has shown synergism with checkpoint inhibition (90, 91). However, clinical proof of this treatment modality has yet to be obtained (89). What is needed first, are better markers of the different activation states so that they can be characterized in patients.

### Targeting TAMs as a Therapeutic Strategy

The recruitment of TAMs into the TME is strongly dependent on the CCL2 and CSF-1 signaling axes mediating their replenishment from circulating monocytes. Thus, multiple treatment strategies including mAbs, small-molecule inhibitors, and RNAi targeting these pathways have been developed (49). In pre-clinical pancreatic cancer models, CSF-1R signaling inhibition reduces both the numbers of tumor-infiltrating TAMs and their expression of immune-suppressive molecules and therefore acts synergistically with checkpoint inhibition (90). In 2017, a promising study reported response to immunotherapy in combination of CSF-1R and PD1 antagonists in pancreatic cancer patients and is now moving on to a phase II clinical trial (64). Conceptually similar, the humanized CSF-1R Ab emactuzumab reduces TAM infiltration and increases T cell infiltration, which was also confirmed in patients with diffuse type giant cell tumors (64). Several CCL2 blockade combination trials are underway and first results showed a 40% increase in chemotherapy response in pancreatic cancer patients (64, 92). Blockade of the CCL2 axis has however limitations, as it is rapidly compensated by granulocytes and cessation of the therapy induces a burst of monocytes from the bone marrow, increasing metastasis and invasion in a breast cancer mouse model, warranting caution (93).

Other than modulating TAM numbers, alternative strategies have focused on directly targeting immune suppressive TAM effector molecules, such as Arg1 inhibitors (94), or on reprogramming TAMs into an anti-tumorigenic population. In murine glioblastoma, inhibition of CSF-1R regressed established tumors and increased survival, which was attributed to a re-education from an M2 to an anti-tumorigenic phenotype (95). Loss of the receptor tyrosine kinase MERTK triggers a proinflammatory TAM phenotype and induces T cell activation (96, 97), while HDAC inhibition reprograms TAMs into highly phagocytic tumor suppressors (91). However, cancer cells can escape phagocytosis by expressing the membrane receptor CD47—the “don't eat me signal,” which binds to SIRPα on macrophages, inhibiting phagocytosis. Several clinical compounds targeting this suppressive axis are currently in clinical trials (98). Despite many encouraging results, TAM targeting still needs further investigation, since it was recently shown that classical monocytes and macrophages are required for better response to checkpoint inhibition in mouse models (99, 100) and that binding of antibodies to FC receptors of macrophages contributes to the success of several therapeutic responses (101). Thus, a depletion strategy specific for pro-tumorigenic TAMs or strategy to convert TAMs is needed, further highlighting the need to identify specific markers.

## Myeloid-Derived Suppressor Cells

Soluble factors released into systemic circulation can cause a differentiation block in normal hematopoiesis and promote the expansion of immature myeloid precursors (IMCs), which fail to terminally differentiate. These so-called MDSCs are best characterized in the field of cancer, but also accumulate in infectious diseases, aging or obesity. They are distinct to terminally differentiated mature myeloid cells (e.g., DCs and TAMs), yet their distinction from neutrophils is often a topic of controversy. As evident from their name, these pathologically activated cells exhibit strong immune suppressive capacities and are crucial drivers of an immune-suppressive microenvironment.

### MDSCs Are a Heterogeneous Population of Highly Immune Suppressive Cells

Myeloblasts give rise to neutrophils and myeloid-dendritic cell progenitors (MDPs) can specify into monocytes, however, upon tumor mediated instruction these fail to fully mature and form MDSCs (Figure 1). MDSCs arise in response to many tumor-derived factors and are further subdivided into two groups: monocytic (M) MDSCs (LY6G^−^/LY6C^high^), which are morphologically similar to monocytes, and polymorphonuclear (PMN) MDSCs (Ly6G^+^/LY6C^low^), which are morphologically similar to neutrophils (102, 103). The distinction between PMD-MDSCs and neutrophils has proven difficult, as they share cellular origin and many phenotypic and morphological features. Thus, a few reports suggest the use of the term N1 and N2 neutrophils for describing different neutrophil activation states, where N2 refers to a more PMD-MDSC like phenotype (104, 105). While a few advances to delineate these cell types have been made, further knowledge and additional markers are needed to faithfully distinguish them (106).

MDSCs are mobilized from the bone marrow via G-CSF, GM-CSF, or hypoxia, and recruited to the tumor site, where inflammatory mediators such as IL-6, TNF-α, and prostaglandin E2 then further enhance their immune suppressive functions. PMN-MDSCs mainly inhibit T cell functions via production of reactive oxygen (ROS) and nitrogen species (NOS), inducing T cell apoptosis or anergy, and do so in an antigen-specific manner. Their relatively weak suppressive role has led to speculations about their contribution to immune suppression. However, their high prevalence in cancer patients and several reports showing improved immune responses upon PMN-MDSCs depletion in mouse models, indicate that further investigation is required to delineate their role. In contrast to PMD-MDSCs, M-MDSCs are considered more suppressive and inhibit both antigen-specific and non-specific T cell responses (1, 78, 106). They exert their suppressive functions via high expression of Arg1, driving T cell anergy by depleting arginine pools (80, 81). In addition, MDSCs can express high levels of IL-10 and TGF-β, and produce reactive nitrogen species, negatively affecting T cell recruitment and activation (1, 78, 107). They also harbor tumor-promoting functions that are independent of immune suppression, such as the promotion of metastasis and angiogenesis via the production of VEGF, bFGF, and MMP9 (Figure 2) (2, 108).

### MDSC Levels Correlate With Poor Patient Prognosis and Resistance to Immunotherapy

In lung, breast, and colorectal cancer the abundance of MDSCs in the tumor has been correlated with advanced stage and decreased overall survival (2), also circulating MDSCs negatively influence patient outcome (109, 110). Circulating neutrophils in clusters with cancer cells were recently reported to promote cell cycle progression and metastatic potential in mouse models and patients (111). While high levels of neutrophils are often associated with poor clinical outcome, they can also have anti-tumorigenic functions, especially in early-stage, small-sized tumors, where they are capable of stimulating T cell responses and secreting proinflammatory mediators. Larger, more advanced tumors preferentially recruit immune suppressive MDSCs (104, 112, 113), which negatively correlates with immunotherapy response in melanoma (110, 114). In conclusion, despite difficulties to faithfully distinguish between PMD-MDSCs and neutrophils, these studies indicate that neutrophils can be both pro- and anti-tumorigenic, whereas MDSCs are exclusively supportive of tumor progression (115).

### Targeting MDSCs as a Therapeutic Strategy

MDSCs can be modulated in several ways, by targeting (1) their formation in the bone marrow, (2) their recruitment to the tumor site, or (3) their immune suppressive activities. For targeting MDSC formation and inhibiting their expansion, all-trans retinoic acid was shown to differentiate MDSCs into mature DCs and macrophages and confirmed to reduce numbers of circulating MDSCs in patients (116, 117). MDSC formation is also reduced as an advantageous side-effect of several cancer cell-targeting therapies, such as the tyrosine kinase inhibitor sunitinib, via blockade of VEGF, and c-kit signaling (118, 119), or the cytotoxic drugs gemcitabine and 5-fluorouracil that induce selective apoptosis of MDSC in several tumor models, while leaving T cells, DCs, B cells and NK cells unharmed (120, 121). In order to inhibit the recruitment of MDSCs to the tumor, targeting of the CCL2 axis is being evaluated. Conceptually similar, antagonists for CCR5 are known to reduce MDSC recruitment (122). Targeting of effector functions can be achieved via inhibition of phosphodiesterase, reducing expression of Arg1 and iNOS (123, 124), similar to the HDAC inhibitor entinostat, which reduces expression of COX2, Arg1, and NOS2 in mouse models of melanoma and renal carcinoma (125). HDAC inhibition was shown to act synergistically with PD-1 inhibition in murine models and clinical trials are underway (126, 127). siRNA or decoy nucleotides targeting the TF STAT3, which drives the immune suppressive activities of MDSCs, represent another therapeutic approach to block immune suppressive features (122). Overall, targeting MDSCs is conceptually very attractive due to the wide range of immune suppressive effector molecules. However, due to their heterogeneous nature and the lack of highly specific surface markers, it remains a challenging task that requires further investigation.

## Perspectives and Outlook

The recent success of T cell targeting agents has validated immune-cell based therapies as an innovative approach to treat cancer. However, immune suppressive mechanisms hampering their success are manifold and myeloid cells are crucial mediators of the suppressive TME. They are a heterogeneous population of cells and rapidly adapting their phenotype to the surrounding tissue. Tumors provide a unique, complex milieu with distinct oncogenic drivers, altered metabolism, hypoxia, and many secreted factors that drive the emergence of myeloid phenotypes unique to the disease. This induces a very complex situation, with many different cell types and activation states that need to be characterized in-depth to allow an understanding of their contribution to immunotherapy success and the development of new therapeutic tools. Technical advances and high dimensional-analytic tools with single cell resolution, ranging from sequencing to mass cytometry, now give us the opportunity to investigate these cell types at an unprecedented rate of detail during steady-state and disease conditions and in different phases of therapy. These tools need to be implemented in immune-oncology in both, patient samples from different cancer entities with clinical follow-up data available, and pre-clinical models that allow their perturbation and experimental testing of therapeutic targets. Together this will allow a deeper understanding of how activation state, localization, and phenotype of myeloid cells in the tumor shape the microenvironment and provide the basis for modulating the tumor microenvironment in targeted approaches, ultimately improving therapeutic outcomes of cancer patients treated with immunotherapy.

## Author Contributions

LH and AO researched the data for article, contributed to the discussion of the content, wrote the manuscript, and reviewed and/or edited the manuscript before submission. The authors apologize that due to space limitations, only selective original articles could be cited.

### Conflict of Interest

The authors declare that the research was conducted in the absence of any commercial or financial relationships that could be construed as a potential conflict of interest.
